# An optimized cocktail of small molecule inhibitors promotes the maturation of dendritic cells in GM-CSF mouse bone marrow culture

**DOI:** 10.3389/fimmu.2023.1264609

**Published:** 2023-10-13

**Authors:** Shintaro Matsuba, Hiroki Ura, Fumiji Saito, Chie Ogasawara, Shigetaka Shimodaira, Yo Niida, Nobuyuki Onai

**Affiliations:** ^1^ Department of Immunology, Kanazawa Medical University, Uchinada, Ishikawa, Japan; ^2^ Center for Clinical Genomics, Kanazawa Medical University Hospital, Uchinada, Ishikawa, Japan; ^3^ Division of Genomic Medicine, Department of Advanced Medicine, Medical Research Institute, Kanazawa Medical University, Uchinada, Ishikawa, Japan; ^4^ Department of Regenerative Medicine, Kanazawa Medical University, Uchinada, Ishikawa, Japan; ^5^ Center for Regenerative Medicine, Kanazawa Medical University Hospital, Uchinada, Ishikawa, Japan

**Keywords:** dendritic cells, small molecule inhibitor, cytokine production, mixed lymphoid reaction, immunotherapy

## Abstract

Dendritic cells (DCs) are the most potent antigen-presenting cells, playing an essential role in the pathogen and tumor recognition, and anti-tumor immunity, and linking both the innate and adaptive immunity. The monocyte-derived DCs generated by ex vivo culture, have been used for cancer immunotherapy to eliminate tumor; however, the clinical efficacies are not sufficient, and further improvement is essential. In this study, we established a method to generate DCs using small molecule compounds for cancer immunotherapy. We observed an increase in the percentage of CD11c^+^I-A/I-E^high^ cells, representing DCs, by adding four small molecular inhibitors: Y27632, PD0325901, PD173074, and PD98059 (abbreviated as YPPP), in mouse bone marrow (BM) culture with granulocyte-macrophage colony stimulating factor (GM-CSF). BM-derived DCs cultured with YPPP (YPPP-DCs) showed high responsiveness to lipopolysaccharide stimulation, resulting in increased interleukin (IL) -12 production and enhanced proliferation activity when co-cultured with naïve T cells compared with the vehicle control. RNA-seq analysis revealed an upregulation of peroxisome proliferator - activated receptor (PPAR) γ associated genes increased in YPPP-DCs. In tumor models treated with anti-programmed death (PD) -1 therapies, mice injected intratumorally with YPPP-DCs as a DCs vaccine exhibited reduced tumor growth and increased survival. These findings suggested that our method would be useful for the induction of DCs that efficiently activate effector T cells for cancer immunotherapy.

## Introduction

Dendritic cells (DCs) initiate the acquired immunity to eliminate cancer and pathogens through antigen presentation and cytokine production in lymphoid and nonlymphoid tissues (1). In the peripheral tissue, DCs capture the debris from tumor, pathogen-infected cells, or dead cells, process them and presents to T cells as antigen. This is important for initiating adaptive immune responses against a variety of antigens, including pathogens, tumors, and self-antigens.

DCs originate from hematopoietic stem cells (HSCs) through several stages of DC progenitors, known as common DC progenitors (CDPs) (2–4). Specific transcription factors for DC polarization have been identified for each type, including conventional DCs (cDCs) and plasmacytoid DCs (pDCs) (2, 5). The cDCs are further subdivided into conventional DC1 (cDC1) and conventional DC2 (cDC2), spread in the lymphoid and non-lymphoid tissues. The cDC1 have excellent cross-presentation activity, therefore, these cells are an attractive target for vaccination against intracellular pathogens and tumors, unfortunately, very few of these cells can be isolated from tissue (6, 7).

Large numbers of DC can be generated from the BM cells as BM-derived DCs using an *in vitro* culture with granulocyte-macrophage colony stimulating factor (GM-CSF) or Fms like tyrosine kinase 3 ligand (Flt3L) (8–10). *In vitro* cultures with GM-CSF mainly produce DCs that are similar to monocyte-derived DCs (8). In contrast, cultures with Flt3L best reflects physiological DC development, but results in a mixture of pDC and cDC, in which cDCs correspond to cDC1 and cDC2 (10). Recently, an efficiently induction culture system for CD103^+^ DCs derived from mouse BM using a combination of GM-CSF and Flt3L has been reported (11). Taking advantage of these features of cDCs, priming tumor antigens to DCs and returning them to the body to induce cancer-specific immune responses is a typical immune-cell therapy (12, 13). However, current methods often fail to obtain the necessary number of DCs from cancer patients for cancer immunotherapy. Previous studies have attempted to induce DCs derived from human embryonic stem (hES) cells or induced pluripotent stem (iPS) cells, but issues such as a long culture time for DC induction, high costs, and the risk of cancerous transformation still persist (14–16).

Recently, it has been reported that a cocktail of low molecular weight compounds can reprogram hepatocytes lacking the proliferative potential into liver progenitor cells with proliferative and differentiation potential (17). While DCs can be induced from the BM or peripheral blood using GM-CSF or Flt3L in an *in vitro* culture system (8, 10), the induction of DCs using by small molecule cocktails has not been reported. Previous studies have shown that inhibition of Rho-associated kinase (ROCK) by the small molecule compound Y27632 can overcome the massive cell death associated with hES cell dissociation (18). Additionally, the mitogen-activated protein kinase (MEK) inhibitors PD98059 and PD0325901 (19–21) has been found to help hES cells survive in culture and maintain their proliferation over time. PD173074, an inhibitor of the fibroblast growth factor (FGF) receptor, has been implicated in hES cell self-renewal (19). Therefore, we hypothesized that these small molecules could efficiently induce high-quality DCs.

This study is the first time to report that a small molecule cocktail, comprising Y27632, PD0325901, PD173074, and PD98059 (YPPP), promotes DC maturation in mouse BM culture with GM-CSF. BM-derived DCs culturing with YPPP (YPPP-DCs) showed heightened responsiveness to lipopolysaccharide (LPS) stimulation, resulting in increased interleukin (IL) -12 production and enhanced proliferation activity of effector T cells. YPPP-DCs showed increased PPARγ-associated gene transcriptions in steady state. These results indicate that YPPP can effectively be used as a DC differentiation and maturation regent.

## Materials and methods

### Mice and reagents

C57BL/6 mice (6-9 weeks old) and BALB/c mice (6-9 weeks old) were purchased from Japan SLC (Shizuoka, Japan). Rag2^-/-^OT-I and Rag2^-/-^OT-II T cell receptor (TCR) transgenic mice (6-9 weeks old) were purchased from Taconic Biosciences. These mice were kept and bred in the animal unit at Kanazawa Medical University, an environmentally controlled and specific pathogen-free facility, in accordance with the guidelines for experimental animals defined by the facilities. Animal experimental protocols were approved by the Animal Research Committee at Kanazawa Medical University (Approval No.:2020-30, 2022-17). The procedures were carried out in accordance with the approved guidelines. Ovalbumin (OVA) _257-264_ peptide SIINFEKL and OVA_323-339_ peptide were purchased from Anaspec.

### Cell lines and culture

E.G7 tumor cell line was purchased from the American Type Culture Collection (ATCC) and B16 melanoma cell line was kindly gifted by Dr. Nobuo Yamaguchi (Kanazawa Medical University). E.G7 cells were cultured in Roswell Park Memorial Institute (RPMI)- 1640 medium modified to contain 2mM L-glutamine, 10mM HEPES, 1mM sodium pyruvate, 4500mg/l glucose, and 1500mg/l sodium bicarbonate (Sigma-Aldrich) supplemented with 10% fetal calf serum (FCS), B16 cells were cultured in Dulbecco’s modified eagle medium (DMEM)supplemented with 10% FCS.

### Preparation of small molecule cocktails

The four small molecule inhibitors (YPPP) were first reconstituted into stock solutions; 10 mM Y27632 (Fujifilm Wako) was prepared in sterilized phosphate buffered saline (PBS), and 40 mM PD0325901 (Fujifilm Wako), 10 mM PD173074 (Fujifilm Wako) and 10 mM PD98059 (Fujifilm Wako) were prepared in dimethyl sulfoxide (DMSO, Nakarai tesque). Stock solutions were stored at −20°C until use. Y27632, PD0325901, PD173074 and PD98059 were used at final concentrations of 50, 0.04, 0.01, 6.3 μM.

### Generation of murine BM-derived dendritic cells

BM cells were prepared from C57BL/6 mice and these cells were cultured with GM-CSF as we described previously (8). Briefly, BM cells were cultured in RPMI-1640 supplemented with 10% FCS, 5 x 10^-5^M 2-mercaptoethanol, 100 U/ml penicillin, 100 μg/ml streptomycin and 25 ng/ml GM-CSF (Biolegend) and with DMSO or YPPP for 6 days. On day 6, CD11c^+^ cells were isolated using the magnetic-activated cell sorting (MACS) system with magnetic microbead-conjugated anti-CD11c antibody (Miltenyi Biotec). The purities of the sorted CD11c^+^ fractions by using CD11c microbeads were consistently ≥90% (**Supplementary Figure 2**). In E.G7 tumor model, CD11c^+^ cells were stimulated with 10 ng/ml lipopolysaccharide (LPS) for 12 h and loaded with 10 μM OVA_257-264_ peptide SIINFEKL for 2h prior to injection. In B16 melanoma model, CD11c^+^ cells were stimulated with 10 ng/ml lipopolysaccharide (LPS) for 12 h prior to injection.

### Flow cytometry and cell sorting

Flow cytometry was conducted using the following antibodies (All purchased from Biolegend unless stated otherwise): anti-CD11c (N418), anti-I-A/I-E (M5/114.15.2), anti-CCR7 (4B12), anti-CD40 (3/23), anti-CD11b (M1/70), anti-CD80 (16-10A1), anti-CD86 (GL-1), anti-CD4 (GK1.5), anti-CD8 (53-6.7), anti-CD25 (PC61), anti-CD62L (MEL14), anti-CD69(H1.2F3) and anti-PD-L2 (TY25). Fc-mediated nonspecific staining was blocked with anti- CD16/32 (2.4G2 hybridoma culture supernatant). Events were acquired using a FACSCanto II (BD Biosciences), and the data of 10,000-100,000 events were analyzed using the FACSDiva (BD Biosciences) or FlowJo software programs (FlowJo). The surface molecule expressions were calculated by defining the delta mean fluorescence intensity between the specific antibody stain and the isotype-matched control antibody. CD11c^+^I-A/I-E^high^ cells and CD11c^+^I-A/I-E^int^ cells were isolated by using a cell sorting system (SH800; Sony Biotechnology) or FACS Aria fusion (BD Biosciences). Separation of these cells were shown in **Supplementary Figures 5A, B**. Briefly, BM lineage negative (Lin (–)) cells were immunomagnetically pre-enriched using PE-Cy5-conjugated antibodies against lineage antigens, including CD3ϵ (145-2C11), CD4 (GK1.5), CD8α (53-6.7), B220 (RA3-6B2), CD19 (MB19-1), CD11c (N418), CD11b (M1/70), Gr-1 (RB6-8C5), NK1.1 (PK136), anti-Cy5 microbeads and autoMACSpro (Miltenyi Biotec). BM- Lin (-) cells were cultured with 25 ng/ml GM-CSF (Biolegend) for 6 days. On day 3, BM-Lin (-) derived CD11c^+^ cells were isolated using the MACS system with magnetic microbead-conjugated anti-CD11c antibody (Miltenyi Biotec). BM- Lin (-) cells were then stained with FITC-anti-I-A/I-E (M5/114114.15.2) and PE-Cy7-anti-CD11c (N418) (all from BioLegend). CD11c^+^I-A/I-E^high^ cells and CD11c^+^I-A/I-E^int^ cells were sorted by gating on indicated cells from CD11c^+^ cells. The purity of the sorted populations was consistently ≥90%.

### *In vitro* T cell proliferation assays

#### OT-I T cells

Thy1.2^+^ T cells from spleen of OT-I or OT-I x Rag2^-/-^ mice were enriched using the MACS system with magnetic microbead-conjugated anti-CD90.2 antibody (Miltenyi Biotec), according to manufacturer’s instructions. Enriched Thy1.2^+^ T cells were then labeled with celltracker Cytotell green (AAT Bioquest) at 37 ˚C for 10 min, washed, and counted before culture with antigen presenting cells (APCs).

#### OT-II T cells

Thy1.2^+^ T cells from spleen of OT-II x Rag2^-/-^ mice were enriched using the MACS system with magnetic microbead-conjugated anti-Thy1.2^+^ antibody (Miltenyi Biotec), according to manufacturer’s instructions. Enriched Thy1.2^+^ T cells were then labeled with celltracker Cytotell green at 37 ˚C for 10 min, washed, and counted before culture with APCs.

#### BALB/c T cells

Thy1.2^+^ T cells from spleen of BALB/c mice were enriched using the MACS system with magnetic microbead-conjugated anti-CD90.2 antibody (Miltenyi Biotec), according to manufacturer’s instructions. Enriched Thy1.2^+^ T cells were then labeled with celltracker Cytotell green at 37°C for 10 min, washed, and counted before culture with APCs.

### Stimulation

Sorted CD11c^+^ cells in mouse bone marrow culture with GM-CSF and DMSO/YPPP for 6 days were incubated in 96-well round-bottom tissue culture plates. For OT-I T cells stimulation, CD11c^+^ cells were incubated with OVA_257-264_ peptide (1 nM) in the presence of 100 ng/ml of LPS. For OT-II T cells stimulation, CD11c^+^ cells were incubated with OVA_323-339_ peptide (10 μM) in the presence of 100 ng/ml of LPS and co-cultured with naive Cytotell green-labeled OT-II T cell. For allogenic BALB/c T cells stimulation, CD11c^+^ cells were incubated with LPS (100 μg/ml). Various number of CD11c^+^ cells (7.3 x, 22.0 x or 66.0 x 10^2^ cells were incubated with 2.0 x 10^4^ T cells. T cell proliferation and activation was assessed by flow cytometry after 3 days for OT-I T cells and after 5 days for OT-II and BALB/c T cells.

### Cell counting kit-8 assay

Cells were grown into 24 well plates. At day 6, 10 μl of CCK-8 solution (Dojindo) was added into each well and cultured for another 2 hours, and measured at 490 nm in a microplate reader Multiscan JX (Thermo scientific). The ratios of proliferation were calculated as follows: the absorbance of culture supernatants at 490 nm/absorbance of the control cell supernatants at 490 nm.

### ELISA

Cells were incubated with the indicated stimulators. The levels of cytokines in the culture supernatants were determined using ELISA kits, in accordance with the manufacturers’ instructions. The mouse IL-12p70, IL-12p40, IL-6, TNF-α ELISA MAX Standard kits (Biolegend) were used.

### Immunoblotting

The cells for immunoblotting were prepared as we described previously (22). Briefly, Cells were solubilized in lysis buffer (1.0% NP-40, 50 mM HEPES, pH7.4, 150 mM NaCl), containing protease and phosphatase inhibitor (Thermo fisher scientific). Cell lysates were separated by sodium dodecyl sulfate (SDS)- polyacrylamide gel electrophoresis (PAGE), transferred to a Polyvinylidene difluoride (PVDF) membrane (Merck), and detected with the following antibodies using ECL substrate (Bio-Rad): rabbit anti-CCAAT enhancer binding protein (C/EBP)α anti-phospho-C/EBPα (Ser21), anti-phospho- C/EBPα (Thr221/226), anti-Erk1/2, anti-phospho- Erk1/2, anti-JNK, anti-phospho-JNK, anti-NF-κB(p65), anti-phospho-NF-κB(p65), mouse anti-IκBα (Cell Signaling Technology), mouse anti-IκBβ (Santa Cruz Biotechnology), mouse anti-βactin (Sigma-aldrich), horseradish peroxidase (HRP)-conjugated mouse IgG (Cell Signaling Technology), or rabbit IgG antibodies (Cell Signaling Technology). Digital images were obtained using an ImageQuant LAS4000 mini instrument (GE Healthcare). Densitometry was performed on scanned blots using the ImageQuant TL software program (GE Healthcare).

### Tumor model

The protocol by which E.G7 or B16 melanoma tumor model were previously described (23, 24). These tumor cells were established by intradermally injecting 2 x or 10 x 10^5^ tumor cells (in 50 μl of DMEM) into the back flanks of 8-9 weeks old C57BL/6 mice (day 0). For dendritic cell vaccine (DCV) therapy, on days 7, 10, 13, 1.25 or 12.5 x 10^4^ syngeneic BM derived CD11c^+^ cells (in 50 μl DMEM/0.1% bovine serum albumin) were injected intratumorally with or without anti Programmed death (PD)-1 (Biolegend, 20μg/mouse) in 200 μl saline intraperitoneal injection. Tumor sizes were measured twice a week. Tumor volume was calculated by the following formula: tumor volume. 0.4 x length (mm) x [width (mm)]^2^ (24). Mice were euthanized when they became moribund or when their tumors exceeded 20 mm in diameter.

### Gene expression analysis

#### Total RNA extraction

Total RNA was extracted with AllPrep DNA/RNA kit (Qiagen) according to the manufacturer’s instructions. Subsequently, the concentration and purity of isolated RNA molecules were measured spectrophotometrically by Nanodrop (Thermo scientific), after which the RNA integrity number was measured using TapeStation 4200 with a High Sensitivity RNA Screen Tape (Agilent Technolgies).

### RNA-seq library construction and library sequencing

The fragmented double-strand cDNA was synthesized using NEB Next Ultra II Directional RNA Library Prep Kit (New England BioLabs) according to manufacturer’s instructions. After library quality was assessed using the TapeStation 4200 with High Sensitivity D1000 ScreenTape (Agilent Technologies). All libraries were quantified using the HS Qubit dsDNA assay (Thermo Fisher Scientific). All libraries were sequenced (2 × 75 bp) using Illumina NextSeq 500 (Illumina) according to the standard Illumina protocol. The FASTQ files were generated using the bcl2fastq software (Illumina).

### Data analysis

FASTQ files were aligned to the reference mouse genome (mm10) using HISAT2 (version 2.1.0) (25). The StringTie algorithm (v.1.3.4d) (26) was then used with default parameter settings to assemble RNA-Seq alignments into annotated transcripts to estimate their expression using the UCSC annotated mouse genome (mm10) assembly file. Subsequently, the transcript expression was normalized using the transcripts per million (TPM) algorithms. For differential expression analysis, we used the R package (edger) (27), as described previously (28, 29). Gene ontology (GO) and Kyoto Encyclopedia of Genes and Genomes (KEGG) analyses were performed using Enrichr web server (https://maayanlab.cloud/Enrichr/) (30–32).

### Statistical analysis

Statistical analysis was carried out by ANOVA using GraphPad Prism software. All experimental repetitions and numbers of specimens and mice are indicated in the figure legends. One-way and two-way ANOVA were used for the experiments with one variable and two variables, respectively. *p* < 0.05 was considered to be statistically significant. For survival analysis, p values were computed using the Log Rank test. p values < 0.05 was considered to be statistically significant.

## Results

### Establishment of the optimal combination of small molecules for DC differentiation

**Figure 1:**
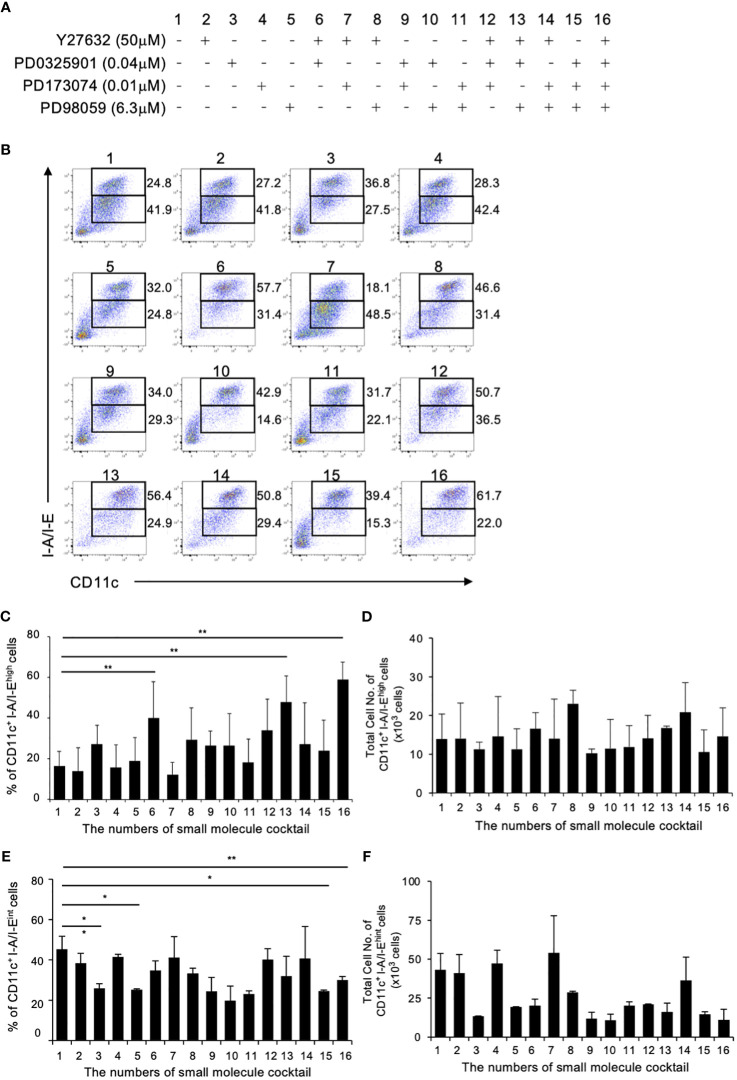
Initial combination trial of four small molecule inhibitors. Using single concentrations of each small molecule cocktail, a total of 16 unique combinations were tested in GM-CSF mouse BM culture. BM cells were cultured with GM-CSF and indicated small molecule cocktail for 6 days. On day 6, Cells were analyzed by flow cytometry. **(A)** The combinations of small molecule cocktail. **(B)** The percentages of CD11c^+^I-A/I-E^high^ cells (Upper fraction) and CD11c^+^I-A/I-E^int^ cells (Lower fraction) on day 6. Data are representative of three independent experiments. **(C, D)** Frequency **(C)** and total cell number **(D)** of the indicated CD11c^+^I-A/I-E^high^ cells (GM-DCs). **(E, F)** Frequency **(E)** and total cell number **(F)** of the indicated CD11c^+^I-A/I-E^int^ cells (GM-Macs). **(C–F)** Data are shown as the mean ± SEM of three independent experiments. *p<0.05, **p<0.01.

Previous study in mouse BM culture with GM-CSF have demonstrated the presence of primarily two subsets: CD11c^+^I-A/I-E^high^ cells (GM-DCs) and CD11c^+^I-A/I-E^int^ cells (GM-Macs) (33). To determine the optimal combination for DC differentiation in a mouse BM-derived DC culture system, we initially examined 16 different combinations of the four small molecule compounds (**Figure 1A**). The optimal concentration of Y27632, PD0325901 and PD173074 were determined through preliminary tests (**Supplementary Figures 1A, B**), while effective concentration of PD98059 was demonstrated in previous study (21, 34). In this culture system, the addition groups of the 6th, 13th, and 16th combination of the small molecule inhibitors significantly increased the percentages of CD11c^+^I-A/I-E^high^ cells in the 40.1 ± 25.0, 44.8 ± 16.5, and 53.4 ± 11.7% compared to 17.4 ± 10.5% for the control (**Figures 1B, C**). No effect was observed with the other additional groups. On the other hand, the addition groups of the 3th, 5th 15th, and 16th combination of the small molecule inhibitors significantly decreased the percentages of CD11c^+^I-A/I-E^int^ cells in the 25.8 ± 2.40, 25.2 ± 0.49, 24.5 ± 0.57, and 30.0 ± 1.77% compared to 45.4 ± 6.35% for the control (**Figures 1B, E**). The total cell number of CD11c^+^I-A/I-E^high^ cells shows no significant difference between control and 16th group (**Figure 1D**). However, the total cell number of CD11c^+^I-A/I-E^int^ cells of 16th group is markedly reduced when compared with control (**Figure 1F**). These results indicate that the addition of these small molecule inhibitor cocktails significantly altered the percentage of CD11c^+^I-A/I-E^high^ cell, representing DCs, with the 16^th^ small molecule inhibitor cocktail YPPP, yielding cells of the highest purity (**Figures 1B-F**). It has been reported that GM-CSF induced differentiation of myeloid-derived suppressor cells (MDSCs) from BM cells, defined as Ly6G^high^Ly6c^int^ cells (35). Therefore, we examined the effect of YPPP on GM-CSF induced MDSCs. The YPPP did not have a significant impact on MDSCs differentiation (**Supplementary Figures 2A, B**).

### Enhanced IL-12 production in YPPP- CD11c^+^ cells after LPS stimulation

**Figure 2:**
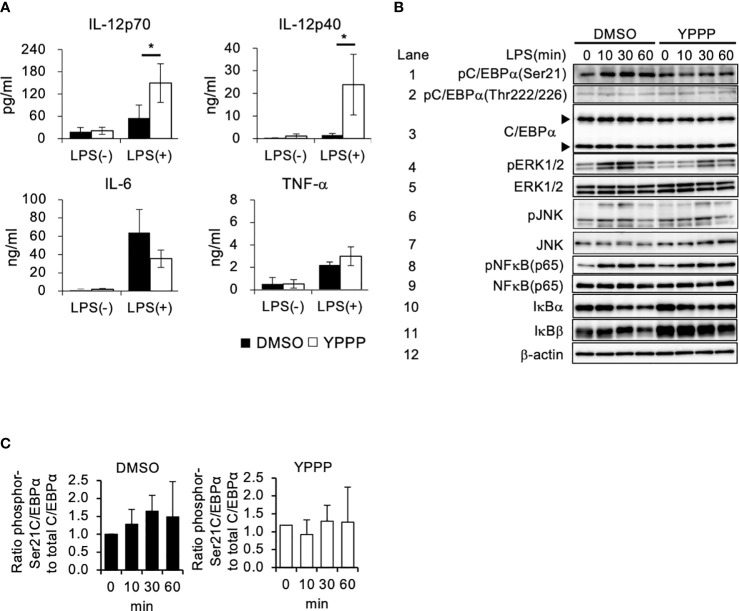
BM-derived CD11c^+^ cells induced by GM-CSF and YPPP increase interleukin (IL) -12 production via the negatively regulation of Toll-like receptor (TLR) 4-mediated C/EBPα activation. **(A)** The IL-12p70, IL-12p40, IL-6 and TNF-α levels in supernatants of DMSO- or YPPP-BM-derived CD11c^+^ cells after LPS stimulation for 48 h. The levels were measured by an ELISA. **(B)** BM-derived CD11c^+^ cells induced by DMSO and YPPP were stimulated with LPS (1μg/ml). Representative immunoblots of the indicated proteins are shown at the indicated time after LPS stimulation. The arrows indicate the 42kDa and 30kDa isoforms of C/EBPα. β-actin was used as loading and internal monitoring controls. **(C)** The relative intensities of phosphor-C/EBPα to total CEBP/α in BM-derived CD11c^+^ cells induced by DMSO and YPPP were estimated by densitometric scanning with normalization to β-actin (means ± SD). Data are representative of two or three separate experiments. *p<0.05.

The anti-tumor activity of DCV used in cancer immunotherapy relies on the quality of the DC-containing cell fraction CD11c^+^ cells in the DCV. Therefore, we examined cytokine production in DMSO- and YPPP-treated BM-derived CD11c^+^ cells, as LPS is known to be a potent inducer of the Th1 cytokine IL-12 by using magmatically purified CD11c^+^ cells (**Supplementary Figure 3**). The level of proinflammatory cytokine IL-6 and IL-12 were measured after LPS stimulation. As shown in **Figure 2A**, YPPP-treated BM derived CD11c^+^ cells (YPPP- CD11c^+^ cells) produced high amount of IL-12p70 and IL-12p40 compared to DMSO-treated CD11c^+^ cells (DMSO- CD11c^+^ cells) after LPS stimulation, while no difference was observed in IL-6 and TNF-α levels between DMSO and YPPP-CD11c^+^ cells. Next, we investigated the activation of key signaling molecules in the Toll like receptor (TLR) 4-mediated signaling pathway, which was activated by LPS stimulation. Previous studies have shown that CCAAT enhancer binding protein alpha (C/EBPα), whose activation is regulated by extracellular signal-regulated kinase 1/2 (Erk1/2), negatively regulates IL-12 production upon LPS stimulation (36). Therefore, we examined the phosphorylation of Erk1/2 and C/EBPα (murine Ser21 and Thr222/226) upon LPS stimulation. While NF-κB and JNK phosphorylation were comparably increased in DMSO- and YPPP-CD11c^+^ cells, Erk1/2 phosphorylation was decreased in YPPP-CD11c^+^ cells (**Figure 2B**, lanes 4, 5, 6, 7, 8 and 9). Surprisingly, pSer21 C/EBPα was decreased in YPPP-CD11c^+^ cells, whereas C/EBPα (Ser21) was phosphorylated in DMSO-CD11c^+^ cells, even after LPS stimulation (**Figure 2B**, lanes 1 and 3, and **Figure 2C**). In contrast, Thr222/226 C/EBPα was equally phosphorylated in both DMSO- and YPPP-CD11c^+^ cells (**Figure 2B**, lane 2). IκBα and IκBβ levels were equivalently decreased in DMSO- or YPPP-CD11c^+^ cells (**Figure 2B**, lanes 10 and 11). These results suggest that IL-12 production is enhanced by suppressing C/EBPα activation upon LPS stimulation in YPPP-CD11c^+^ cells.

### YPPP-CD11c^+^ cells primed ovalbumin are more efficient in T cell activation and proliferation than DMSO-CD11c^+^ cells

**Figure 3:**
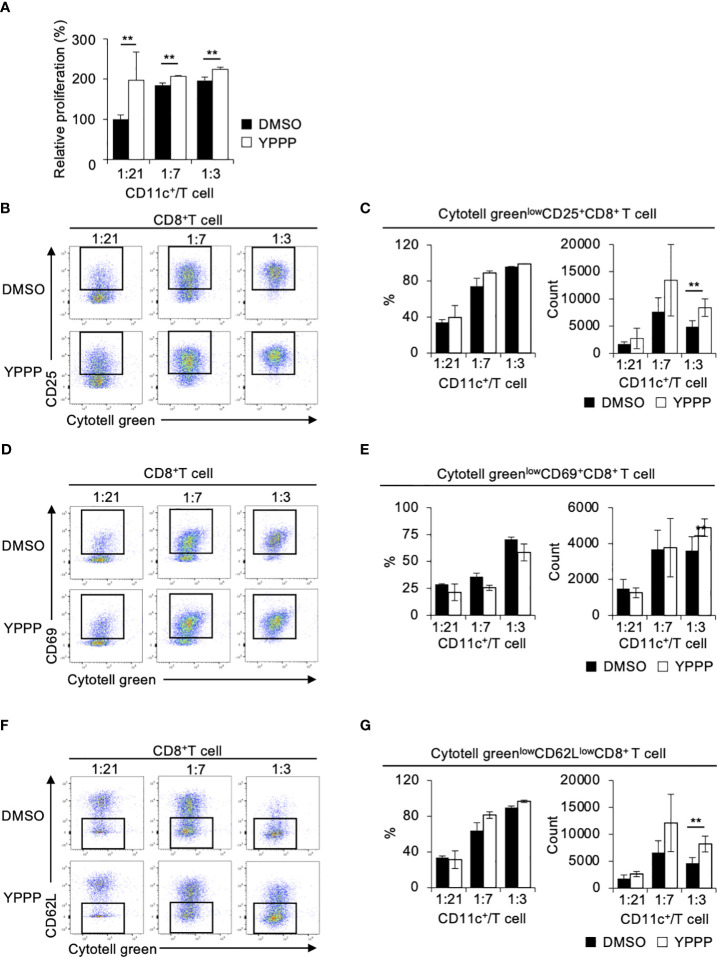
BM-derived CD11c^+^ cells induced by GM-CSF and YPPP augments the activation of OT-I T cells *in vitro*. DMSO- or YPPP-BM-derived CD11c^+^ cells were incubated with OVA_257-264_ peptide and co-cultured with Cytotell green labeled naive splenic Thy1.2^+^ cells. T cell proliferation and expansion were assessed on 3 days after co-cultured. **(A)** T cell proliferation assay measured by Cell Counting Kit 8 (CCK-8). The relative proliferation of OT-I T cells in indicated BM derived CD11c^+^ cells and T cells ratios at day 3 were calculated as the ratio of proliferation to CD11c^+^/T cell **(B, D, F)**. FCM analysis of Cytotell green^low^CD25^+^CD8^+^ T cells **(B)**, Cytotell green^low^CD69^+^CD8^+^ T cells **(D)** and of Cytotell green^low^CD62^low^CD8^+^ T cells **(F)**. **(C, E, G)** Frequency (left) and number (right) of the indicated cells. Data in the bar graph are mean ± SD of triplicate wells for the representative experiment shown. **p<0.01.

The ability of DCV to promote T cell proliferation is a critical function for anti-tumor activity. Therefore, we examined the T cell proliferation activities of DMSO- or YPPP-CD11c^+^ cells in a mixed lymphocyte reaction (MLR). Cell tracker Cytotell green labeled Thy1^+^ splenic CD4^+^ or CD8^+^ T cells from OT-II or OT-I mice were co-cultured with DMSO- or YPPP-CD11c^+^ cells from B6-WT mice for 3 or 5 days *in vitro*. The proliferation was monitored using CCK-8 and flow cytometry. As shown in **Figure 3A**, we observed a significantly increased number of proliferating cells in the group in which YPPP-CD11c^+^ cells, as determined by the CCK-8 proliferation assay. Furthermore, flow cytometric (FCM) analysis revealed that a significant increase of the population of Cytotell green^low^CD25^+^CD8^+^ T cells, Cytotell green^low^CD69^+^CD8^+^ T cells, and Cytotell green^low^CD62L^low^CD8^+^ T cells at 1: 3 CD11c^+^/T cell ratio in the presence of YPPP-CD11c^+^ cells (**Figures 3B-G**).

Next, we examined whether YPPP-CD11c^+^ cells can efficiently expand splenic CD4^+^ T cells isolated from OT-II mice. As shown in **Supplementary Figure 4A**, we found a significantly increased number of proliferating cells in the group in which YPPP-CD11c^+^ cells, as determined by the CCK-8 assay. FCM analysis revealed that an increased population of Cytotell green^low^CD25^+^CD4^+^ T cells and Cytotell green^low^CD69^+^CD4^+^ T cells in co-culture groups with YPPP-CD11c^+^ cells (**Supplementary Figures 4B-E**). Consistent with previous results, the MLR assay using BALB/c T cells also demonstrated enhancement of T-cell proliferation and activation in the presence of YPPP-CD11c^+^ cells (**Supplementary Figures 5A-E**). These results indicate that the YPPP-CD11c^+^ cells are more efficient than DMSO-CD11c^+^ cells in priming and expanding T cells.

**Figure 4:**
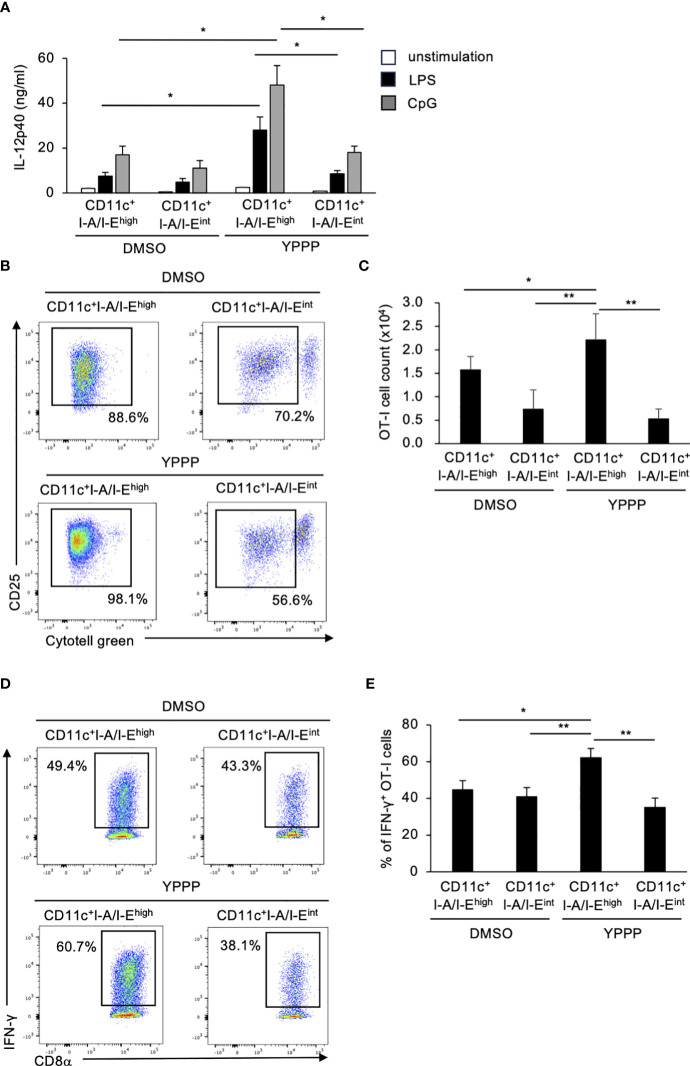
BM-derived CD11c^+^I-A/I-E^high^ cells induced by GM-CSF and YPPP augments IL-12p40 production and the activation of OT-I T cells *in vitro*. **(A)** IL-12p40 levels in supernatants of DMSO- or YPPP-BM-derived CD11c^+^I-A/I-E^high^ and CD11c^+^I-A/I-E^int^ cells after LPS (100 ng/ml) or CpG (1nM) stimulation for 48 h. The levels were measured by an ELISA. **(B, C)** DMSO- or YPPP-BM derived CD11c^+^I-A/I-E^high^ and CD11c^+^I-A/I-E^int^ cells were incubated with OVA_257-264_ peptide and co-cultured with Cytotell green labeled naive splenic Thy1.2^+^ cells. T cell proliferation and expansion were assessed on 3 days after co-culture. FCM analysis of Cytotell green^low^CD25^+^CD8^+^ T cells **(B)** and total cell number of CD25^+^CD8^+^ T cells **(C)**. **(D, E)** The percentage of IFN-γ^+^ cells differentiated from naïve OT-I cells cocultured with DMSO- or YPPP-BM-derived CD11c^+^I-A/I-E^high^ and CD11c^+^I-A/I-E^int^ cells. Data in the bar graph are mean ± SD of triplicate wells for the representative experiment shown. *p<0.05, **p<0.01.

Finally, we compared IL-12p40 production and T cell activation ability between CD11c**^+^** I-A/I-E^high^ cells and CD11c^+^I-A/I-E^int^ cells treated with DMSO/YPPP. The YPPP-treated CD11c^+^I-A/I-E^high^ cells produced more IL-12p40 than the other cells upon LPS or CpG stimulation (**Figure 4A**). Additionally, CD11c**^+^** I-A/I-E^high^ cells demonstrated the highest CD8^+^ T cell proliferation and IFN-γ^+^ cell-inducing-abilities compared with other cells (**Figures 4B-E**).

### Characterization of YPPP-CD11c^+^ cells

**Figure 5:**
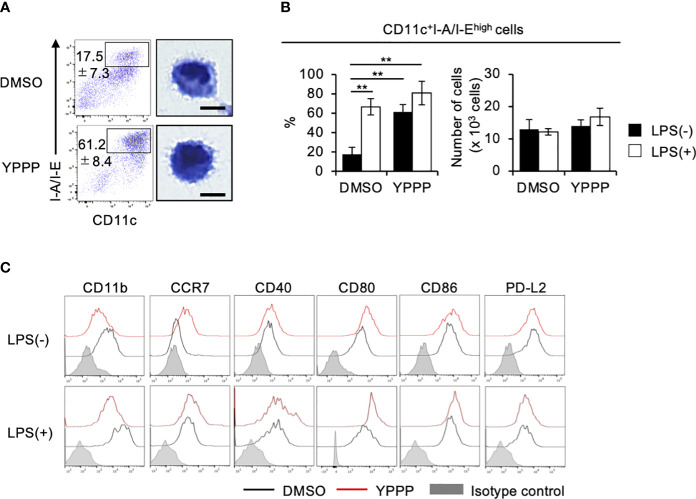
YPPP enhances the DC maturation in mouse BM culture with GM-CSF. **(A)** Representative flow cytometry profiles (left) and microscopic images (right) by Diff-Quick staining of sorted CD11c^+^I-A/I-E^high^ cells in GM-CSF mouse BM culture (Scale bar: 5um). **(B)** Frequency (left) and number (right) of the indicated cells with or without LPS stimulation. **(C)** Representative histograms of the indicated surface antigens from BM-derived CD11c^+^I-A/I-E^high^ cultured with DMSO (black) or YPPP (red). The shaded area shows the isotype control. **p<0.05.

To elucidate the mechanism of enhanced IL-12 production and T cell priming activity in YPPP-CD11c^+^ cells, we examined the effect of YPPP on the differentiation of DCs derived from mouse BM cells cultured with GM-CSF. We focused on the morphological analysis of CD11c^+^I-A/I-E^high^ cells. FCM analysis showed that an increased population of the CD11c^+^I-A/I-E^high^ cells in the YPPP treatment group and a decreased population of CD11c^+^I-A/I-E^int^ cells, representing macrophages (**Figure 5A**; **Supplementary Figures 6A, B**). Diff-Quick staining did show marked differences between DMSO- and YPPP-CD11c^+^I-A/I-E^high^ cells (**Figure 5A**). There were no significant differences in the number of terminally differentiated cells with or without YPPP, and responsiveness to LPS stimulation (**Figure 5B**). Furthermore, FCM analysis of these DC surface makers revealed up-regulation of CD11c, CCR7, and CD80, and down-regulation of CD11b in the YPPP-treated CD11c^+^I-A/I-E^high^ cells (**Figure 5C**). Additionally, both DMSO-treated and YPPP-treated DCs did not express cDC1 marker XCR1 (**Supplementary Figure 7**). These changes align with the general characteristics of GM-DCs (33).

### Gene expression profiles of CD11c^+^I-A/I-E^high^ cells and CD11c^+^I-A/I-E^int^ cells in DC vaccines

**Figure 6:**
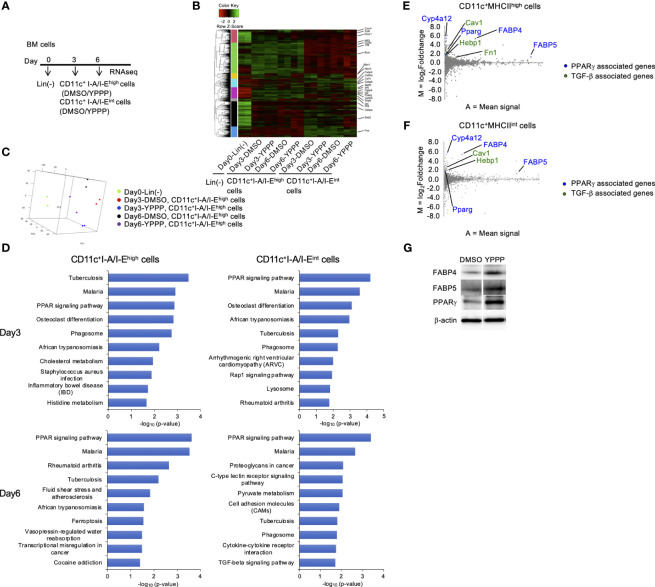
PPARγ-associated genes are increased in BM-derived CD11c^+^I-A/I-E^high^ cells induced by GM-CSF and YPPP. **(A)** Experimental scheme of RNAseq analysis. RNA extracted on day 0, 3 and 6 in GM-CSF mouse BM culture. **(B)** Heatmap of differentially expressed genes of CD11c^+^I-A/I-E^high^ cells and CD11c^+^I-A/I-E^int^ cells in BM derived CD11c^+^ cells. **(C)** PCA analysis of indicated cells. **(D)** Bar chart of top enriched terms from the KEGG_2019_Mouse gene set library. The top 10 enriched terms for the YPPP up-regulated gene set are displayed based on the -log10(p-value), with the actual p-value shown next to each term. The term at the top has the most significant overlap with the input query gene set. **(E, F)** MA plot showing differentially expressed genes in YPPP- versus DMSO-CD11c^+^I-A/I-E^high^ cells **(E)** and CD11c^+^I-A/I-E^int^ cells **(F)** on day 6. Up-regulated PPARγ and TGF-β associated genes are highlighted in the indicated colors. **(G)** Representative immunoblots of the indicated proteins in BM-derived CD11c^+^ cells induced by GM-CSF and DMSO/YPPP.

To compare *in vitro* DC induced by YPPP, we performed RNA sequencing (RNA-Seq) of CD11c^+^I-A/I-E^high^ cells and CD11c^+^I-A/I-E^int^ cells differentiated under DMSO or YPPP conditions for 0, 3, and 6 days (**Figure 6A**). As a result, approximately 6,030, 5,620, 5,626 and 5,662 differentially expressed (DE) genes were identified between DMSO-CD11c^+^I-A/I-E^high^ cells and YPPP-CD11c^+^I-A/I-E^high^ cells on day 3, between DMSO-CD11c^+^I-A/I-E^int^ cells and YPPP-CD11c^+^ I-A/I-E^int^ cells on day 3, between DMSO-CD11c^+^I-A/I-E^high^ cells and YPPP-CD11c^+^I-A/I-E^high^ cells on day 6, and between DMSO-CD11c^+^I-A/I-E^int^ cells and YPPP-CD11c^+^ I-A/I-E^int^ cells on day 6 (**Figure 6B**; **Supplementary Figures 8A-D**). Principal component analysis (PCA) based on differentially expressed genes clearly separated CD11c^+^I-A/I-E^high^ cells cultured with or without YPPP based on both location and genotype (**Figure 6C**). The outcomes of ChIP Enrichment Analysis (ChEA) revealed a significant enrichment of different expressed gene set, and the top 15 enriched gene set up- or down- regulated by YPPP are shown in **Supplementary Figure 8**. And so some of genes known to be important for DC differentiation were shown (**Supplementary Figure 8E**). The outcomes of Kyoto Encyclopedia of Genes and Genomes (KEGG) pathway enrichment analysis revealed a significant enrichment of different expressed gene set, and the top 10 enriched gene set up-regulated by YPPP are shown in **Figure 6D**. Within this gene set, Peroxisome Proliferator-Activated Receptor (PPAR) signaling pathway genes exhibited statistically significant upregulation in YPPP-treated CD11c^+^I-A/I-E^high^ cells when compared to DMSO-treated CD11c^+^I-A/I-E^high^ cells. The MA plot showed up-regulated several genes associated with PPARγ, and transforming growth factor (TGF)-β (**Figures 6E, F**). Consistent with RNA-seq analysis, immunoblotting demonstrated higher expression level of fatty acid binding protein (FABP) 4, FABP5 and PPARγ in YPPP-CD11c^+^ cells compared to DMSO-CD11c^+^ cells (**Figure 6G**). Previous reports have shown that FABP family plays an important role in DC function and T cell priming (37, 38). These results provide further insight in our data that the YPPP-treated CD11c^+^I-A/I-E^high^ cells produced more IL-12p40 than DMSO-treated CD11c^+^I-A/I-E^high^ cells upon LPS and CpG stimulation (**Figure 4A**). Additionally, no significant differences were observed in the expression of the major cDC1-related genes (Xcr1, Irf8, and Batf3) between DMSO- and YPPP-treated cells (**Supplementary Figure 8E**). These results suggested that YPPP instructs the direction of DC differentiation via the activating of PPARγ and TGF-β associated genes.

### The DCV produced by YPPP has an excellent anti-tumor activity

**Figure 7:**
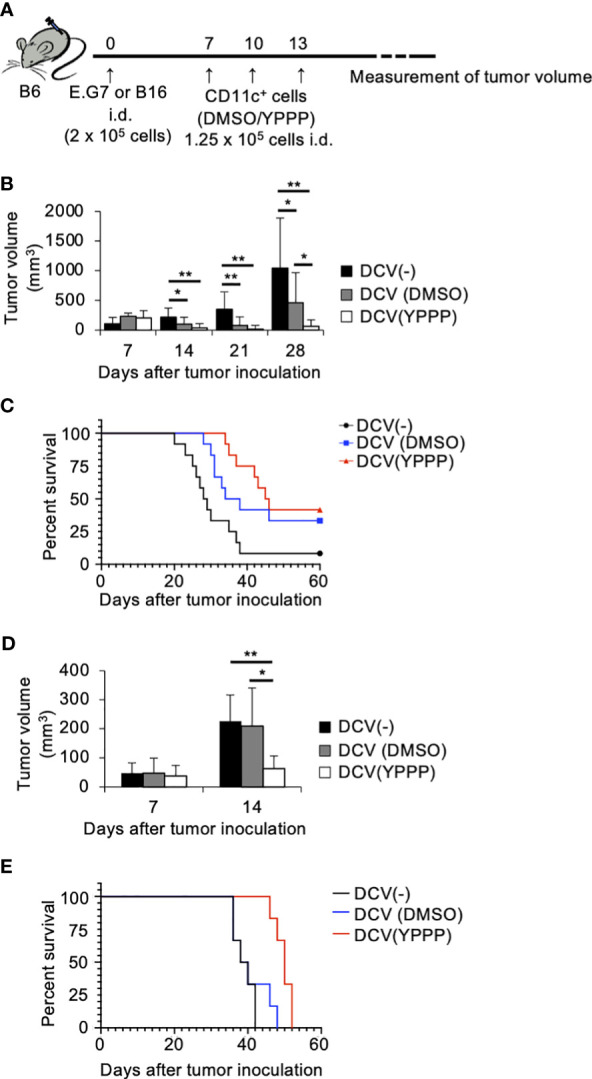
Small molecule cocktail YPPP enhances the anti-tumor activity of dendritic cells vaccine. **(A)** Schematic of tumor vaccine strategy. Mice were injected with 2 x 10^5^ E.G7 or B16 melanoma tumor cells. On the 7, 10 and 13 days after tumor inoculation, mice were vaccinated with 1.25 x 10^5^ BM-derived CD11c^+^ cells induced by GM-CSF and DMSO/YPPP. The growth or rejection of E.G7 or B16 melanoma tumor was monitored. E.G7 **(B)** or B16 melanoma **(D)** tumor volume in untreated (DCV(-)), DMSO-CD11c^+^ cells (DCV(DMSO)) or YPPP-CD11c^+^ cells (DCV(YPPP)) vaccinated mice at indicated days after tumor inoculation. Kaplan –Meier curves of E.G7 **(C)** or B16 melanoma **(E)** showing survival of mice. * *p*<0.05, ** *p*<0.01.

To investigate the *in vivo* anti-tumor potential of the DCV produced by adding YPPP, we evaluated its therapeutic efficacy against established E.G7 and B16 melanoma tumor models (**Figure 7A**). The mice were injected with E.G7 tumor cells (Day 0). On day 7, 10 and 13 post tumor inoculation, the mice were vaccinated with DMSO- or YPPP-CD11c^+^ cells cultured with GM-CSF. As shown in **Figures 7B, C**, a notable decrease in tumor growth and an improvement in survival is apparent within the DCV-administered group (both DMSO and YPPP treated-group), in comparison to the DCV-non-administered group. In addition, DMSO-CD11c^+^ cells (DMSO-DCV) were unable to suppress tumor growth completely, whereas YPPP-CD11c^+^ cells (YPPP-DCV) significantly reduced tumor growth, although they could not eradicate the tumor. These results indicated that YPPP-DCV has superior anti-tumor effects than DMSO-DCV (**Figures 7B-E**).

### YPPP-DCV enhances the efficacy of anti-PD-1 based cancer therapy

**Figure 8:**
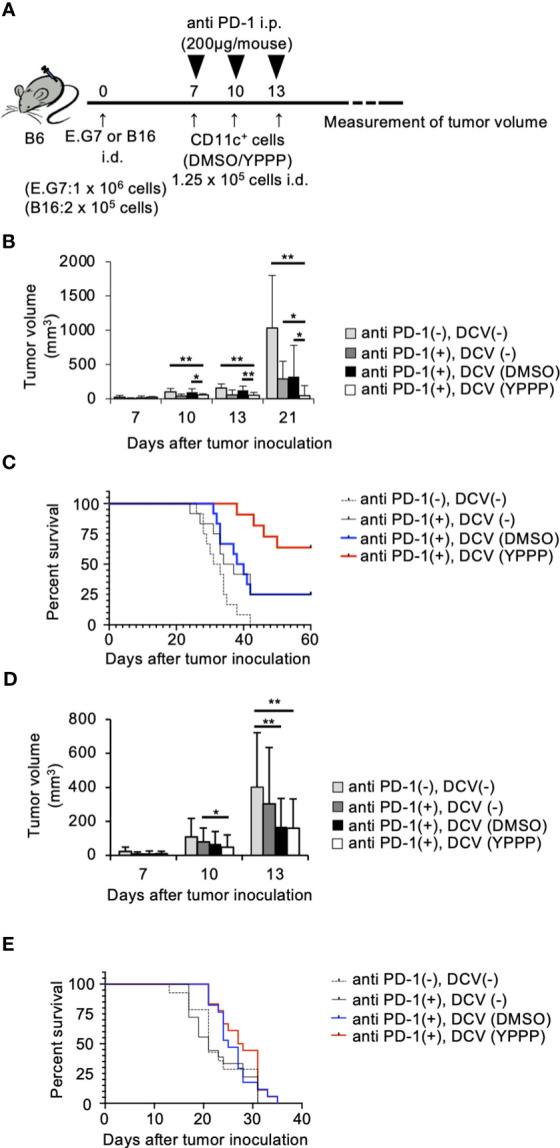
Small molecule cocktail YPPP enhances the anti-tumor activity of dendritic cells vaccine in anti PD-1 therapy. **(A)** Schematic of tumor vaccine strategy. Mice were injected with 1 x 10^6^ E.G7 or 2 x 10^5^ B16 melanoma tumor cells. On the 7, 10 and 13 days after tumor inoculation, mice were vaccinated with 1.25 x 10^4^ BM-derived CD11c^+^ cells induced by GM-CSF and DMSO/YPPP. The growth or rejection of E.G7 or B16 melanoma tumor was monitored. E.G7 **(B)** or B16 melanoma **(D)** tumor volume in untreated (anti PD-1(-), DCV(-)), anti-PD-1 treatment only (anti PD-1(+),DCV(-)), anti-PD-1 and DMSO-CD11c^+^ cells (anti PD-1(+), DCV(DMSO)) or YPPP-CD11c^+^ cells (anti PD-1(+), DCV(YPPP)) vaccinated mice at indicated days after tumor inoculation. Kaplan –Meier curves of E.G7 **(C)** or B16 melanoma **(E)** showing survival of mice. * *p*<0.05, ** *p*<0.01.

The combination of anti-PD-1, an immune checkpoint inhibitor, and other therapies has shown remarkable efficacy (39). Thus, we examined the combined anti-tumor activities of DCV with anti-PD-1 in the same tumor model as mentioned above. As shown in **Figures 8A-C**, tumor growth and mortality of the group treated with YPPP-DCV significantly reduced in the E.G7 tumor model (Log-rank test, p=0.0164), although these activities were not observed in the B16 melanoma model (**Figures 8D, E**). These results suggest that YPPP-DCV enhances the efficacy of anti-PD-1 therapy in some cancer models.

## Discussion

In this study, we screened small molecule inhibitor cocktails and discovered that a particular cocktail, YPPP promoted DC differentiation in GM-CSF mouse BM cultures. DCs induced with YPPP produced more IL-12 upon LPS stimulation, leading to a strong promotion of cytotoxic T cell activation and proliferation. In addition, we found that DCs induced by YPPP had a synergistic effect with anti-PD-1 therapy, resulting in high anti-tumor activity.

Previous report has demonstrated that GM-CSF-induced BMDC consist of two subsets: CD11c^+^I-A/I-E^high^ cells (GM-DC) and CD11c^+^I-A/I-E^int^ cells (GM-Mac). The CD11c^+^I-A/I-E^high^ cells originate from CDP, while CD11c^+^I-A/I-E^int^ cells were derived from common monocyte progenitor (cMoP) and monocytes (33). As shown in **Figure 1B**, the predominant fraction form BM cultured with YPPP is CD11c^+^I-A/I-E^high^ cells when compared with DMSO-control. The total cell number of CD11c^+^I-A/I-E^high^ cells shows no significant difference between control and 16th group. (**Figure 1D**). However, the total cell number of CD11c^+^I-A/I-E^int^ cells of 16th group is markedly reduced when compared with control (**Figure 1F**). Consequently, it is likely that YPPP suppresses the differentiation of cMoP and monocytes into CD11c^+^I-A/I-E^int^ cells, rather than promoting the differentiation from CDP into CD11c^+^I-A/I-E^high^ cells.

Previous reports have demonstrated that a specific ROCK inhibitor, Y27632, is protected from apoptosis in hES cells, and regulates morphology and function in human monocyte-derived DCs (40–42). Moreover, MEK mitogen-activated protein kinase inhibitors, such as PD0325901 and PD98059, are negatively regulates cytokine production in macrophages (43, 44). Furthermore, FGF receptor inhibitor, PD173074, plays an essential role in osteocyte differentiation (44). However, most of these studies focused on short-term administration of small molecule compounds, and the effects of their long-term administration are largely unknown. Additionally, it has been reported that high doses of PD98059 (25μM or 50μM) induce apoptosis in human monocyte derived DCs (45, 46). Conversely, low concentrations of PD98059 (6.3μM) contribute the CD11c^+^I-A/I-E^high^ cells differentiation in GM-CSF-induced BMDC with other small compounds. It is important to note that none of the compounds exhibit any toxicity for BMDCs in our experimental condition. Our data provide novel insights for understanding DC differentiation and activation.

The role of PPARγ in the differentiation and function of myeloid cells has yielded conflicted results (47, 48). The previous study has reported pharmacological inhibition of PPARγ induces differentiation and immunogenic function of human monocyte-derived DCs (48). Our research demonstrated an up-regulation of PPARγ and its-associated genes (FABP4 and FABP5) in the YPPP-treated DCs. These cells exhibited increased capacity for IL-12 production, CD8^+^ T cell proliferation, and induction of IFN-γ^+^ CD8^+^ T cells. Moreover, previous study has reported that both FABP4 and FABP5 play a role in IL-12 production and T cell priming in human monocyte-derived DCs (37, 38). Therefore, the enhancement PPARγ signaling contributes to the increased DC function in the YPPP-treated DC.

We observed strong suppression of phosphorylation of C/EBPα (Ser21) in YPPP-CD11c^+^ cellsafter LPS stimulation, while pSer21 C/EBPα was remarkably increased in DMSO- CD11c^+^ cells. A previous study reported that the phosphorylated C/EBPα (Ser21) inhibits IL-12/IL-23 expression (36), suggesting that YPPP induces high IL-12 production by suppressing C/EBPα activation. Further studies are required to clarify the detailed mechanism how YPPP affects the C/EBP pathway upon LPS stimulation, as YPPP may prevent ERK1/2-dependent C/EBPα activation and impair Ser21 phosphorylation of C/EBPα.

It has been reported that GM-CSF-induced monocyte-derived DC exhibit a restricted ability to migrate during lymph nodes (49). Therefore, the limited effectiveness of the DC vaccine could be a result of protocols that fail to induce an optimal T cell priming. Endogenous DCs are required for T cell priming upon injection of antigen-loaded exogenous DCs (50, 51). In our study, we utilized ova peptide-loaded GM-CSF-induced BMDCs for DC vaccine in E.G7 tumor model. We demonstrated a significant reduction in tumor growth and an improvement in survival within the YPPP-treated DC-administered group. Furthermore, we confirmed that YPPP-treatment led to an up-regulation CCR7 in the DCs, a critical chemokine receptor facilitating the migration of DCs into the lymph node. While both exogenous and endogenous DCs play a role in T cell priming, it is likely that YPPP-treated DCs efficiently migrate lymph node and act as a source of antigen.

The YPPP-treated CD11c^+^ cells were included the CD11c**^+^** I-A/I-E^high^ cells and CD11c^+^I-A/I-E^int^ cells. The YPPP-treated CD11c**^+^** I-A/I-E^high^ cells demonstrated more CD8^+^ T cell proliferation and IFN-γ cell-inducing abilities compare with the YPPP-treated CD11c^+^I-A/I-E^int^ cells. These data demonstrated that YPPP-treated CD11c**^+^** I-A/I-E^high^ cells possesses significantly superior dendritic cell capabilities. Consequently, it is suggested that YPPP-treated CD11c**^+^** I-A/I-E^high^ cells may exbibit and contribute anti-tumor effect *in vivo*.Our data showed that *in vivo* anti-tumor activity of YPPP-DCV was superior to that of DMSO-DCV in E.G7 or B16 melanoma-bearing mouse models. However, our results with YPPP-DCV are consistent with a previous report−the complete elimination of tumor growth was not achieved; therefore, further therapeutic modifications are required to address this issue. The efficacy of DCV in combination with anti-PD-1 therapy has been reported (23, 24). Moreover, the combination of antibodies that block these other inhibitory receptors is expected to enhance the anti-tumor effect of DCV. Our experiments focused in the E.G7 tumor cells, which have shown sensitivity to anti-PD-1 in previous studies (52), and B16 melanoma cells, in which little effect was observed (53). The combined effect of anti-PD-1 and DCV was more prominent for E.G7, especially for YPPP-DCV, showing strong tumor growth suppression and improved survival. However, the anti-tumor activity of YPPP-DCV was limited in the B16 melanoma model, suggesting that the combination therapy with YPPP-DCV and anti-PD-1 provides more effective anti-tumor immunity against anti-PD-1-sensitive cancer cells.

The potential of administration of GM-CSF based therapy to cancer patients has been suggested (54). Therefore, co-administration of YPPP and GM-CSF with anti-PD-1 to tumor-bearing mouse model is expected to be effective. However, the dose of GM-CSF was critical for anti- or pro-tumorigenic effect in human (54). Thus, the fine turning of GM-CSF and YPPP concentrations will be necessary. On the other hand, the YPPP-DCV is a simpler approach. Therefore, we propose development of DCV with small molecule inhibitors, combined with anti-PD-1 therapy to enhance *in vivo* anti-tumor activity. To achieve successful cancer immunotherapy, a more detailed mechanistic understanding of YPPP-DCV and further strategies to overcome tumor immunosuppressive mechanisms will be essential.

## Data availability statement

The datasets presented in this study can be found in online repositories. The names of the repository/repositories and accession number(s) can be found below: https://www.ncbi.nlm.nih.gov; GSE239334.

## Ethics statement

The animal study was approved by the Animal Research Committee at Kanazawa Medical University (Approval No.:2020-30, 2022-17). The study was conducted in accordance with the local legislation and institutional requirements.

## Author contributions

SM: Funding acquisition, Investigation, Formal Analysis, Writing – original draft. HU: Formal Analysis, Investigation, Methodology, Writing – review & editing. FS: Investigation, Writing – review & editing, Data curation. CO: Data curation, Investigation, Writing – review & editing. SS: Writing – review & editing. YN: Writing – review & editing, Formal Analysis, Methodology. NO: Supervision, Methodology, Writing – review & editing, Conceptualization, Funding acquisition, Investigation.
