# Supplementary material for: An optimized cocktail of small molecule inhibitors promotes the maturation of dendritic cells in GM-CSF mouse bone marrow culture
Source: Front Immunol. 2023 Oct 13;14:1264609. doi: 10.3389/fimmu.2023.1264609 (PMC10611476; doi:10.3389/fimmu.2023.1264609)
Supplement: Supplementary file 1 [file Presentation_1.pdf]

Supplementary Figure 1. Matsuba *et al.*

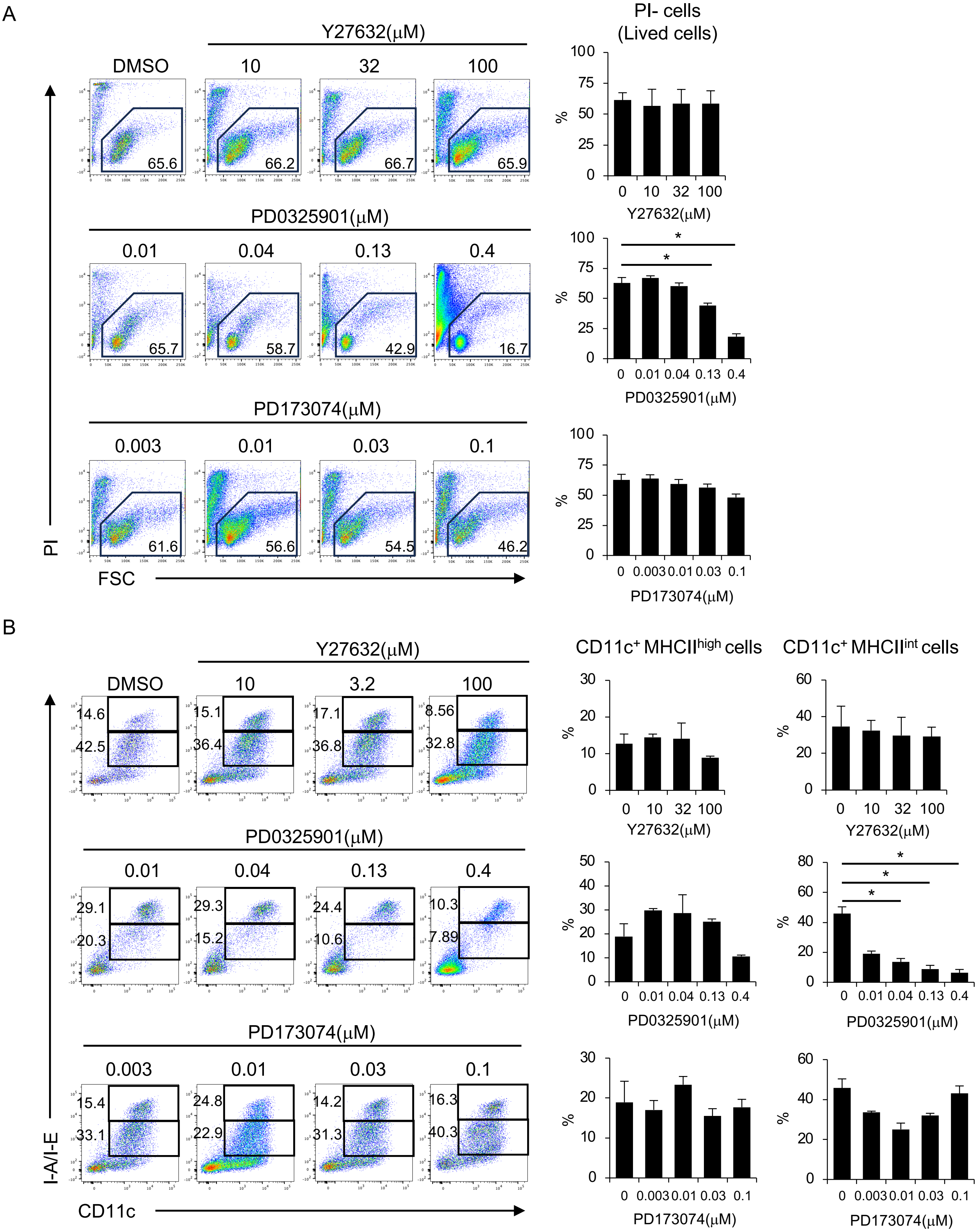

Supplementary Figure 1. Preliminary tests of optimal concentrations of Y27632, PD0325901 and PD173074 for DC induction in GM-CSF mouse BM culture.
BM cells were cultured with GM-CSF and indicated small molecule inhibitor for 6 days. Day 6, Cells were analyzed by flow cytometry. (A) The percentages of PI^- cells (Lived cells). (B) The percentages of $CD11c^+I\text{-}A/I\text{-}E^{high}$ cells (Upper fraction) and $CD11c^+I\text{-}A/I\text{-}E^{int}$ cells (Lower fraction).

A

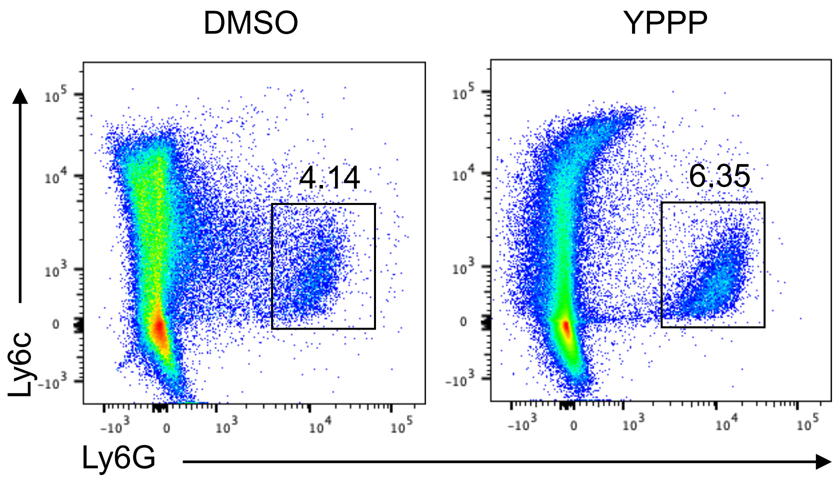

B

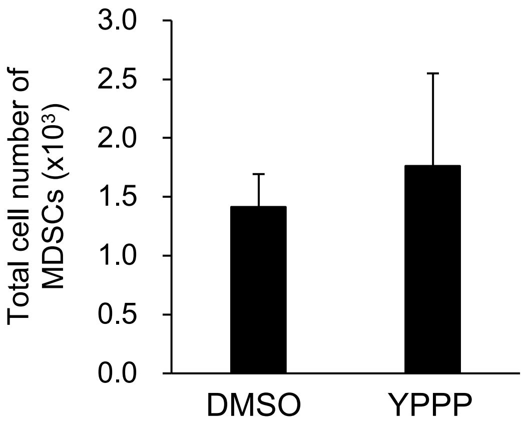

Supplementary Figure 2. The effect of YPPP on MDSCs differentiation. BM cells were cultured with GM-CSF and DMSO/YPPP. (A) The percentage, and (B) total cell number of Ly6G^{high}Ly6c^{int} cells (MDSCs) on day 6. Data are representative of three independent experiments. Data are shown as the mean ± SEM of three independent experiments.

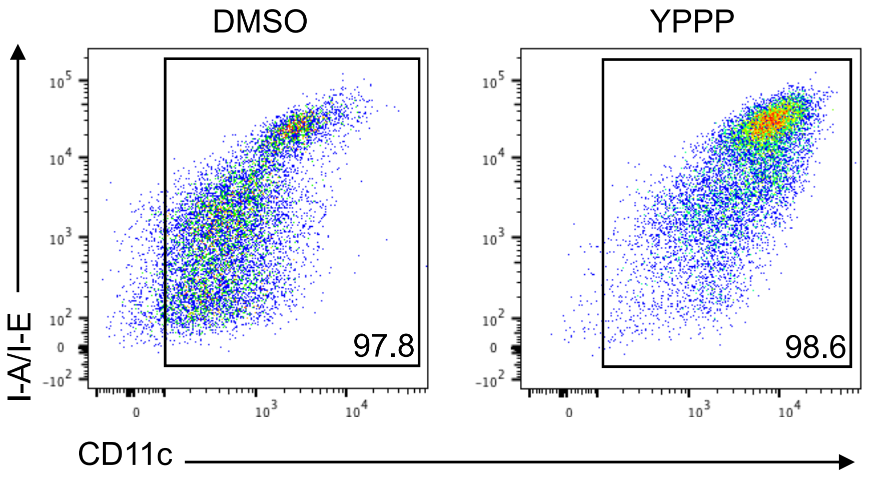

Supplementary Figure 3. FACS analysis of CD11c⁺ cells sorted from BM cells culturing with GM-CSF and DMSO/YPPP for 6 days. Data are representative of three independent experiments.

Supplementary Figure 4. Matsuba *et al.*

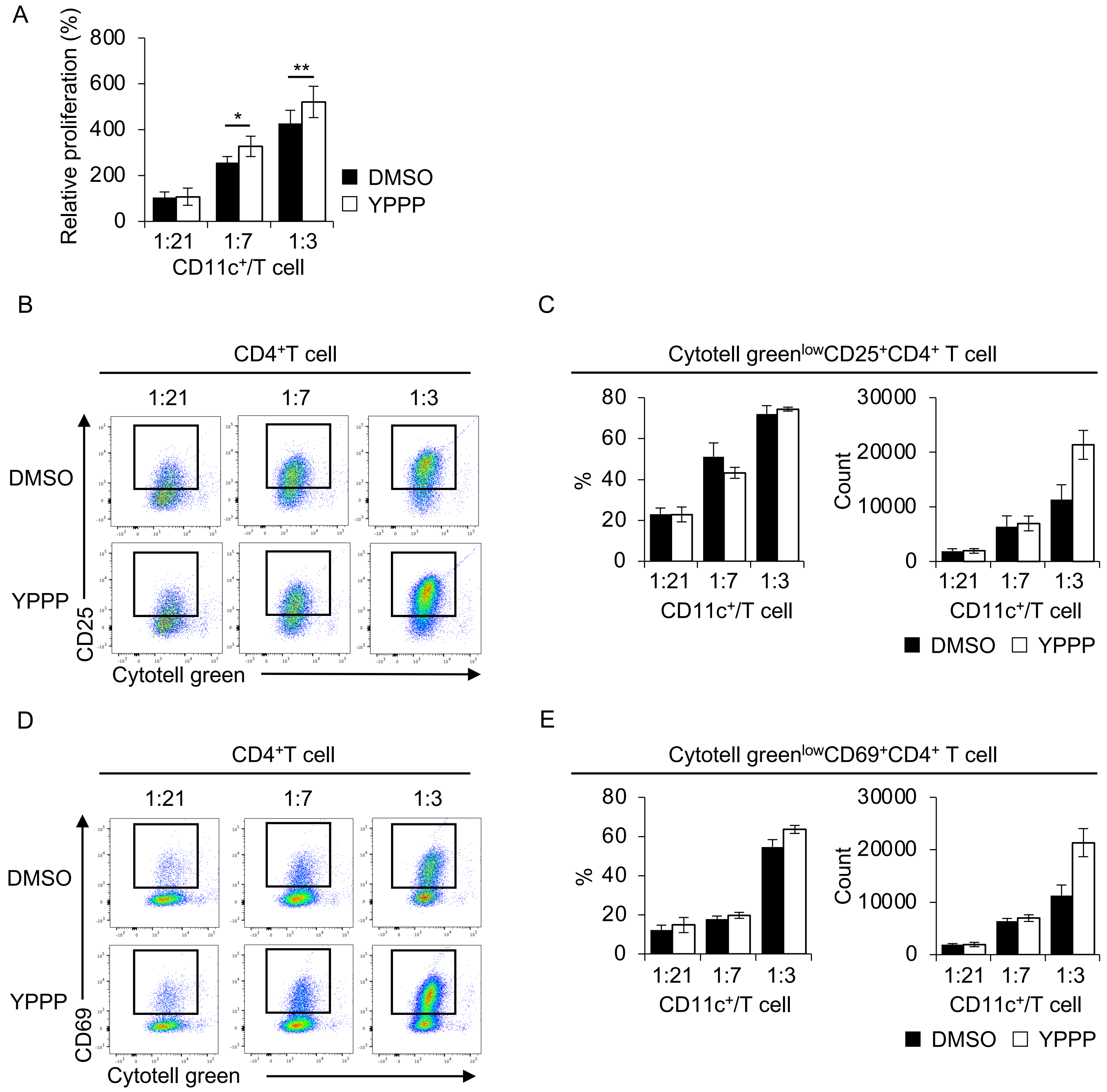

Supplementary Figure 4. BM-derived CD11c^{+} cells induced by GM-CSF and YPPP augments the activation of OT-II T cells in vitro.
DMSO- or YPPP-BM derived CD11c^{+} cells were incubated with $OVA_{323\text{-}339}$ peptide and co-cultured with Cytotell green labeled naive splenic Thy1.2^{+} cells. T cell proliferation and expansion were assessed on 5 days after co-culture. (A) T cell proliferation assay measured by Cell Counting Kit 8 (CCK-8). The relative proliferation of OT-II T cells in indicated BM derived CD11c^{+} cells and T cells ratios at day 5 were calculated as the ratio of proliferation to CD11c^{+}/T cell. (B, D) FCM analysis of Cytotell green^{low}CD25^{+}CD4^{+} T cells (B) and Cytotell green^{low}CD69^{+}CD4^{+} T cells (D). (C, E) Frequency (left) and number (right) of the indicated cells. Data in the bar graph are mean ± SD of triplicate wells for the representative experiment shown. *p<0.05, **p<0.01

Supplementary Figure 5. Matsuba *et al.*

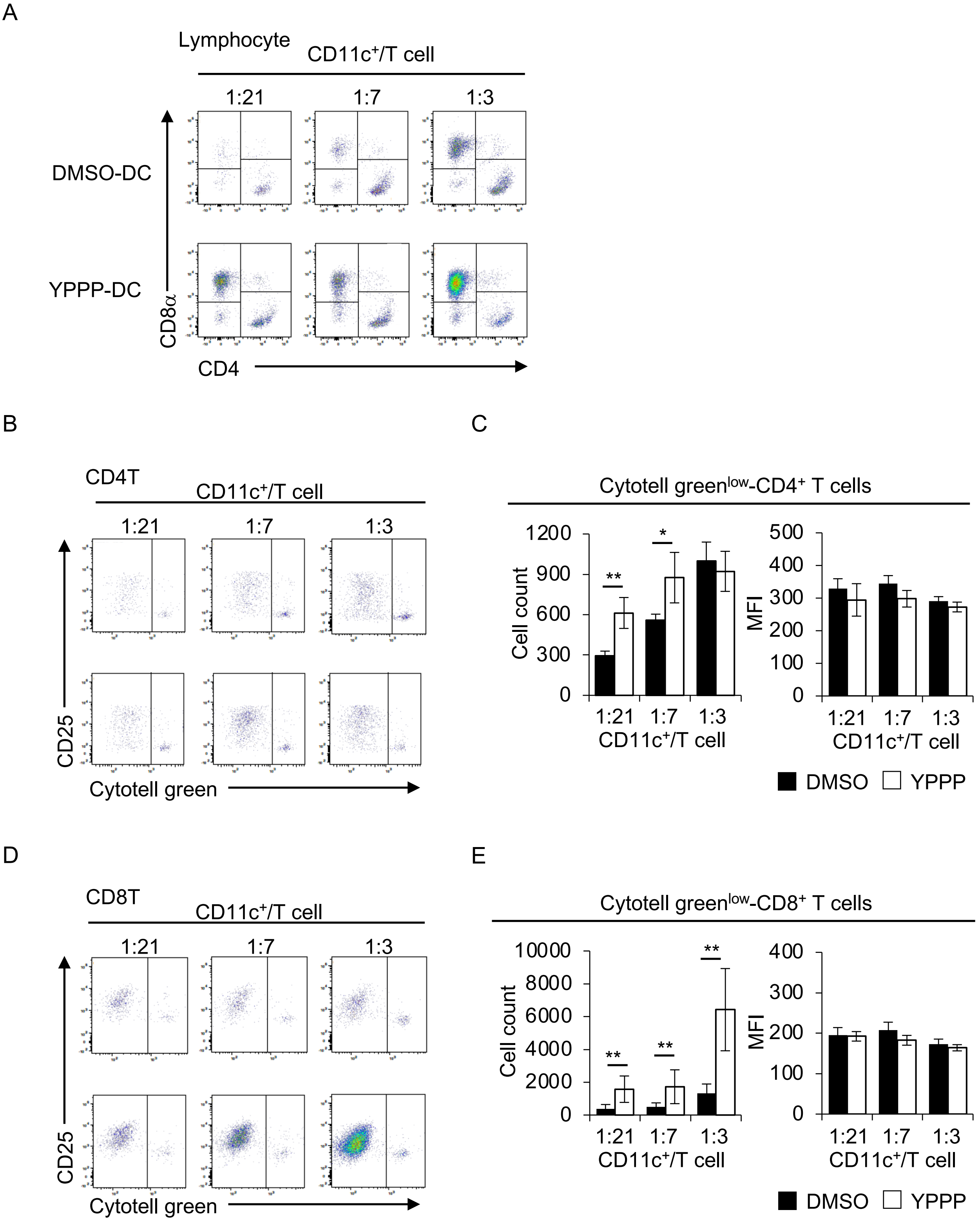

Supplementary Figure 5. BM derived CD11c+ cells induced by GM-CSF and YPPP augments the activation of peripheral BALB/c T cells in vitro.

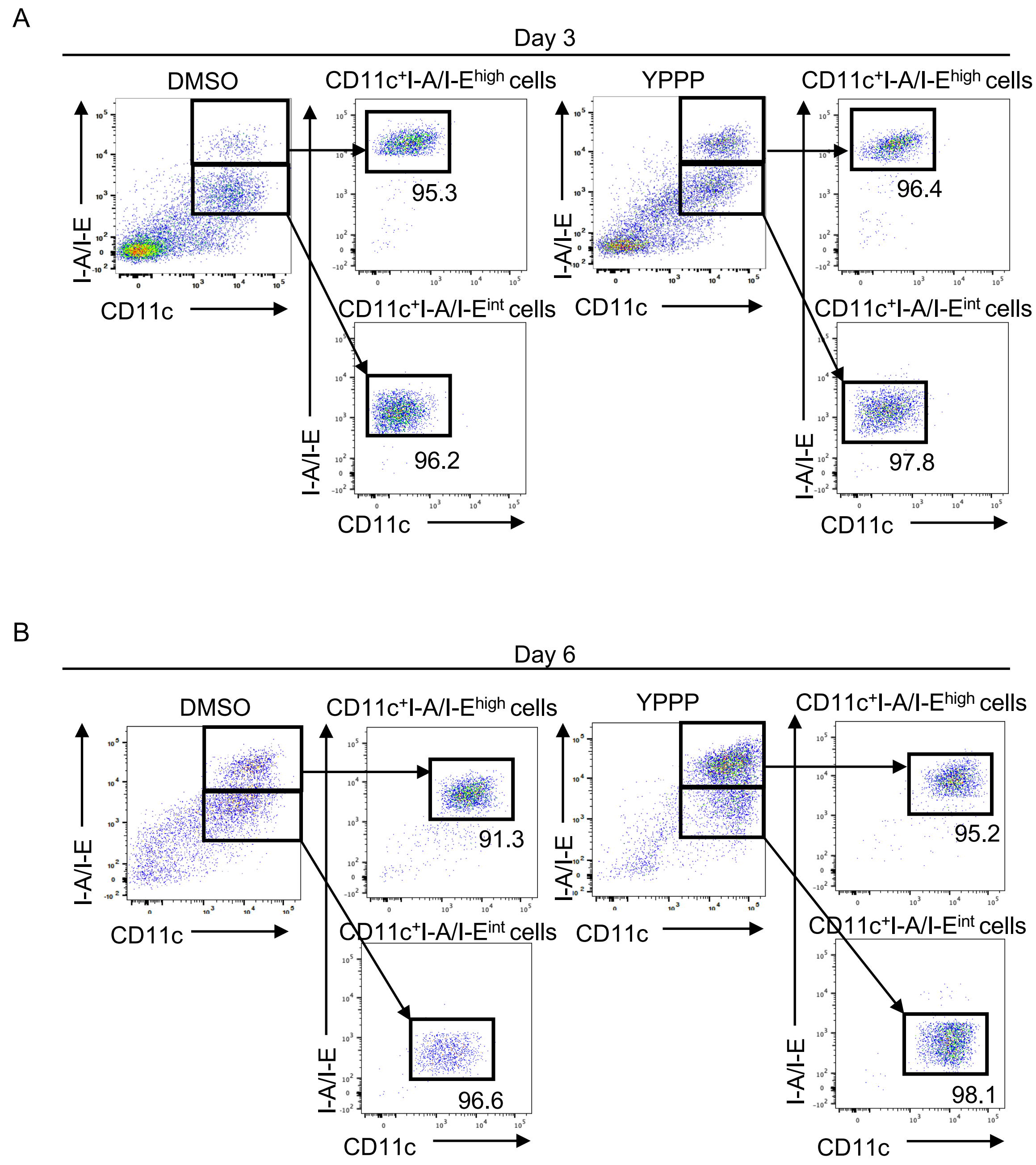

Supplementary Figure 6. CD11c$^+$I-A/I-E^{high} cells and CD11c$^+$I-A/I-E^{int} cells were sorted from BM cells culturing with GM-CSF and DMSO/YPPP for 3 or 6 days (purify≥90%). Upper-left panel shows the expression of CD11c$^+$I-A/I-E^{high} cells and CD11c$^+$I-A/I-E^{int} cells in BM cells culturing with GM-CSF with DMSO for 3 days. Upper-right panel shows the expression of CD11c$^+$I-A/I-E^{high} cells and CD11c$^+$I-A/I-E^{int} cells in BM cells culturing with GM-CSF and YPPP for 3 days. Lower-left panel shows the expression of CD11c$^+$I-A/I-E^{high} cells and CD11c$^+$I-A/I-E^{int} cells in BM cells culturing with GM-CSF with DMSO for 6 days. Lower-right panel shows the expression of CD11c$^+$I-A/I-E^{high} cells and CD11c$^+$I-A/I-E^{int} cells in BM cells culturing with GM-CSF and YPPP for 6 days.

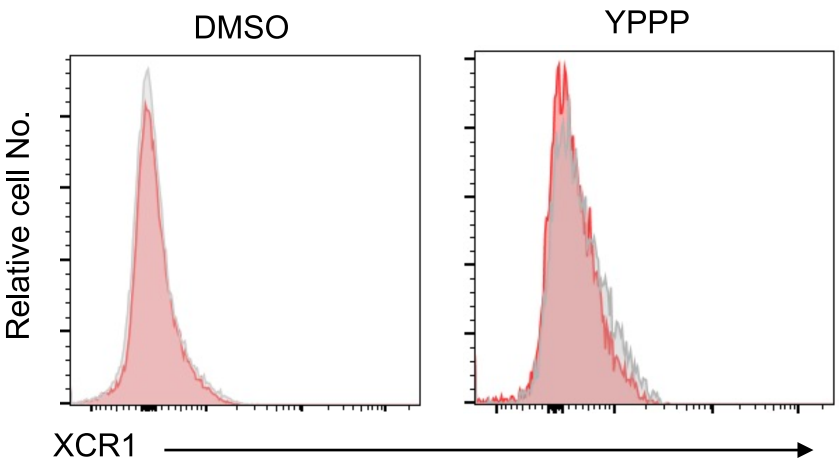

Supplementary Figure 7. Representative histograms of the indicated XCR1 expresion on the DMSO- and YPPP- treated CD11c⁺ cells. The red area shows the isotype control and the gray area shows the XCR1 expression. The data are representative of three independent experiments.

Supplementary Figure 8(1). Matsuba *et al*.

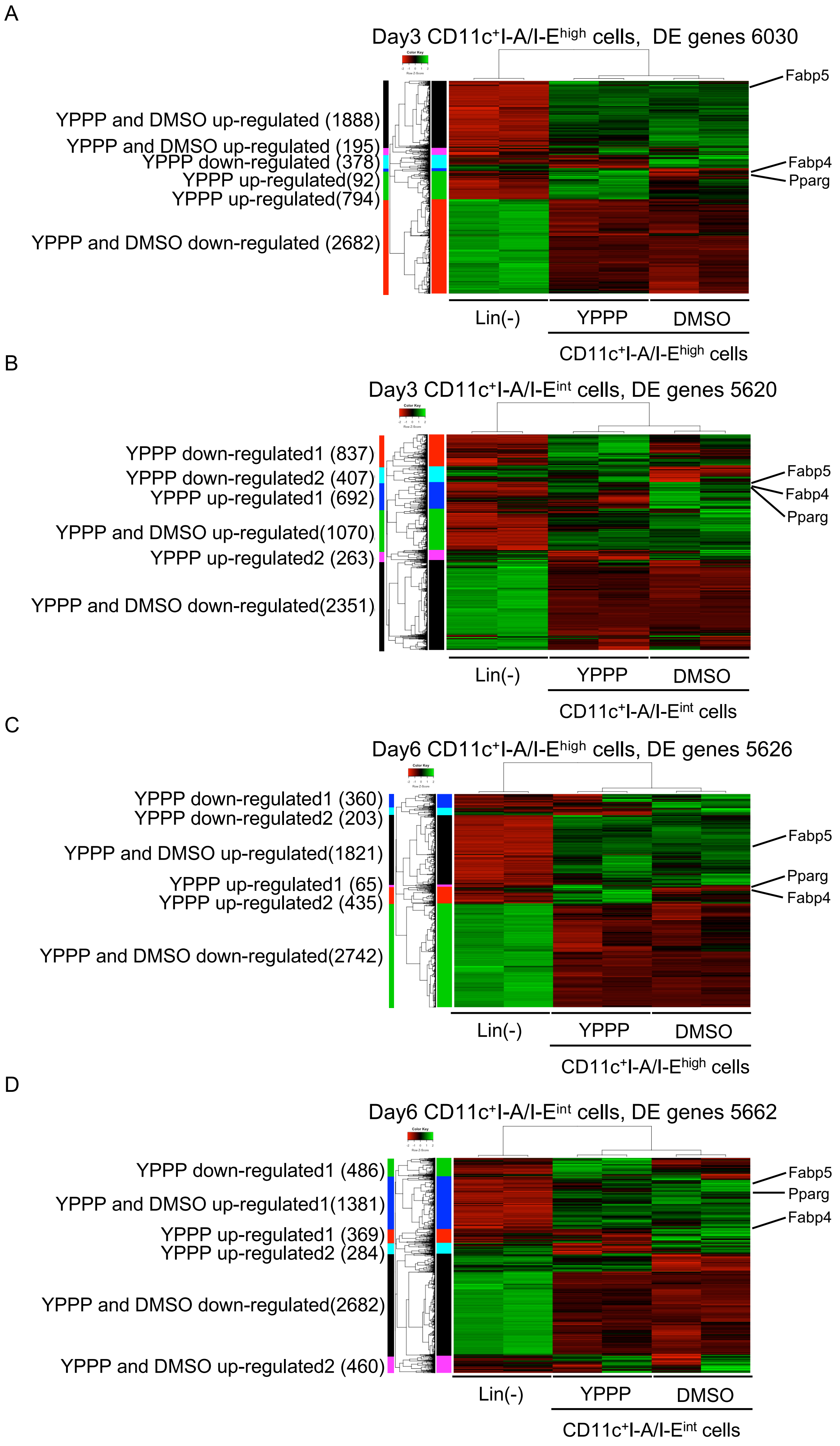

E

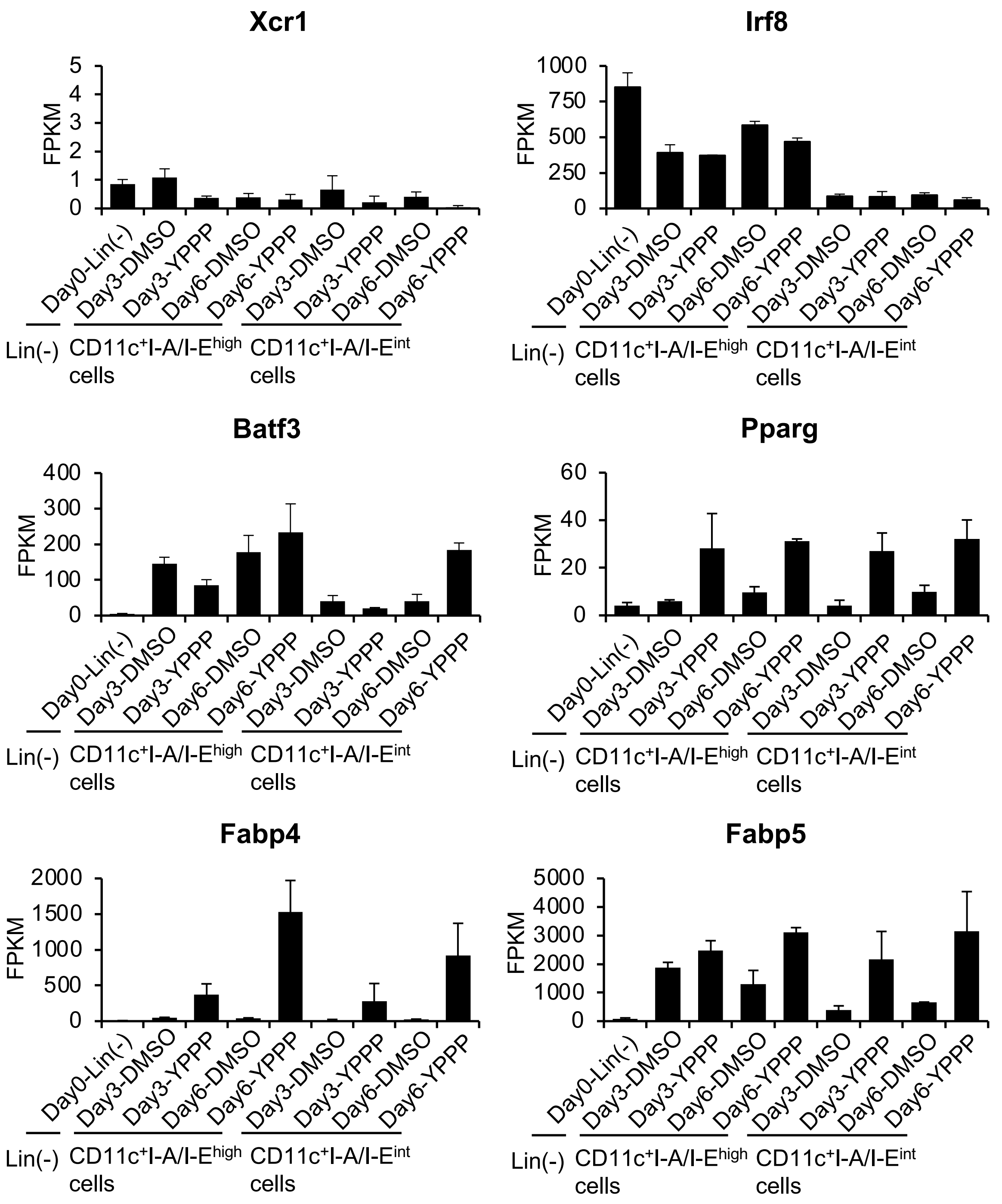

Supplementary Figure 8. RNAseq analysis of CD11c^{+}I-A/I-E^{high} cells and CD11c^{+}I-A/I-E^{int} cells in dendritic cell vaccines.

(A-D) Heatmap of differentially expressed genes of CD11c^{+}I-A/I-E^{high} cells cultured for 3 or 6 days (A, C), and of CD11c^{+}I-A/I-E^{int} cells cultured for 3 or 6 days (B, D). (E) FPKM values obtained by RNA-Seq analysis for indicated genes in relation to the differentiation and the function of conventional DC. Cells were harvested and RNA extracted at 0, 3 and 6 days after polarization. CD11c^{+}I-A/I-E^{high} cells and CD11c^{+}I-A/I-E^{int} cells were derived from BM cells culturing with GM-CSF and small molecules (DMSO or YPPP) for 3 or 6 days. These cells were isolated by fluorescence-activated cell sorter. Clustered heatmap of differentially expressed (DE) genes at day 3 or 6. Color scale is based on normalized read counts.
